# Racial Disparities in the Prescribing of Guideline-Recommended Medications for Metastatic Prostate Cancer: A Retrospective Cohort Study

**DOI:** 10.1155/proc/6500084

**Published:** 2025-11-26

**Authors:** J. N. Stein, A. M. Deal, H. Winslow, K. Morgan, H. Muthukrishnan, Y. E. Whang, M. Charlot

**Affiliations:** ^1^Lineberger Comprehensive Cancer Center, University of North Carolina, Chapel Hill, North Carolina, USA; ^2^Division of Oncology, Department of Medicine, University of North Carolina, Chapel Hill, North Carolina, USA; ^3^UNC School of Medicine, University of North Carolina, Chapel Hill, North Carolina, USA; ^4^UNC Eshelman School of Pharmacy, University of North Carolina, Chapel Hill, North Carolina, USA

## Abstract

**Background:**

Prostate cancer is the second leading cause of cancer death among men, with a disproportionate burden on Black men. Racial disparities in care delivery for early-stage disease are well documented but less is known about racial gaps in advanced prostate cancer care, a stage where effective therapies can prolong life for years. We sought to evaluate potential treatment disparities among Black and White men with metastatic prostate cancer.

**Methods:**

We performed a retrospective cohort study of patients with metastatic prostate cancer receiving treatment at a large public tertiary care health system between 2015 and 2020 using electronic health record data. We estimated the prevalence ratio (PR) of being prescribed each of the recommended treatment options for metastatic prostate cancer as per National Comprehensive Care Network guidelines, including androgen receptor pathway inhibitors (ARPIs) and other antiandrogens, chemotherapy, and bone protection, comparing Black men to White men.

**Results:**

We identified 1166 patients with metastatic prostate cancer treated with androgen deprivation therapy (ADT); 370 (32%) were Black. Prescribing of systemic treatments did not differ by race, notably including ARPI (PR: 0.98 95% CI: 0.98–1.1, *p*=0.8). About 30% of both Black and White patients interacted with our patient navigation team, a group of oncology nurses focused on ensuring patients receive recommended care.

**Conclusions:**

In a large public tertiary care health system, we did not observe racial disparities in the prescribing of guideline-recommended therapies for metastatic prostate cancer. High rates of insurance, a robust patient navigation program, and a well-developed pharmacy assistance program may have helped mitigate racial disparities in care. Future studies should prospectively evaluate the delivery of prostate cancer therapies across health systems and the influence of navigation and pharmacy assistance programs.

## 1. Introduction

Racial disparities in prostate cancer are among the most pronounced in any malignancy. Black men are 1.7 times more likely to develop prostate cancer than their White counterparts and 2.1 times more likely to die of the disease [1]. In high-risk, early-stage disease, there are substantial disparities in the delivery of recommended care, as Black men are less likely to receive definitive treatment than White men [2], less likely to undergo radical prostatectomy [3], and less likely to receive a pelvic lymph node dissection even when they do receive surgery [4]. Black men experience longer wait times from diagnosis to treatment initiation, regardless of the modality selected, even after adjustment for sociodemographic and clinical factors [5]. While there are some indicators that the prevalence of aggressive disease is higher among Black men compared to White men, numerous studies have identified that the vast majority of the disparity in mortality is attributable to gaps in access and care delivery [6, 7]. In advanced disease, a recent nationwide SEER-Medicare analysis found that Black men are less likely to receive all types of treatment, including radiation, chemotherapy, androgen deprivation therapy (ADT), and antiandrogens [8]. These findings are troubling and undermine the goal of equity in cancer care, an essential characteristic in high-quality healthcare as defined by the National Academy of Medicine [9]. However, that analysis is the only published study to our knowledge that explores racial gaps in care delivery for advanced disease.

We sought to characterize prescribing of evidence-based guideline-recommended medications for advanced prostate cancer at a large public tertiary care health system using electronic health record (EHR) data. Our focus was to explore potential racial disparities in care delivery between Black and White men with metastatic prostate cancer. We hypothesized that Black men would be less likely to be prescribed guideline-recommended systemic therapies.

## 2. Methods

### 2.1. Sample

We identified a retrospective cohort of Black and White men receiving cancer treatment within a large public tertiary care health system in the southeastern US from 2015 to 2020 using EHR data. Inclusion criteria were as follows: presence of ICD codes consistent with metastatic prostate cancer diagnoses using a previously validated algorithm [10] and documented visits with oncology providers at one of the seven sites of care: at the main, comprehensive cancer center or one of the affiliate sites. Affiliate sites included six cancer centers distributed across the state, five rural and one urban. Inclusion criteria also required evidence of ADT medication prescribing after the date that an ICD9 or 10 code, indicating metastatic disease. was applied. This ensured patients had chosen to receive medical management for their advanced prostate cancer within the health system. As many patients come to tertiary care systems for second opinions but receive their care locally, we chose to exclude patients without any prostate cancer medication prescribing as we wanted to limit our cohort to patients for whom we had complete prescribing data capture. Exclusion criteria were as follows: lack of ICD9 or 10 codes, indicative of secondary metastatic disease; race other than Black or White; and lack of visits with oncology providers during the study time period.

### 2.2. Outcomes

Medications of interest included 6 categories of therapy: androgen receptor pathway inhibitors (ARPIs), other antiandrogens, bone protection, poly (ADP-ribose) polymerase (PARP) inhibitors, chemotherapy, and others (such as radiopharmaceuticals), based on National Comprehensive Cancer Network (NCCN) and American Society for Clinical Oncology (ASCO) prostate cancer guidelines for systemic therapy in the metastatic setting [11, 12]. Within these categories, there are multiple ARPIs, other antiandrogens, bone protection agents, and chemotherapy regimens (Supporting Table [available here]).

All medication data were pulled for the cohort, including medications received prior to metastatic diagnosis and up to 2 years after metastatic diagnosis. For each drug type, patients were considered to have been prescribed the medication in the metastatic setting if they were on it at the time of metastatic diagnosis, or it was started any time after.

### 2.3. Covariates

We extracted demographic data from the EHR including age, race, ethnicity, marital status, insurance status, and zip code to determine regional income and educational achievement. We also extracted clinical characteristics and other treatments (surgery, radiation, and cryosurgery), the Charlson Index score, primary diagnosis date, as well as interactions with our patient navigation (PN) team, which is largely delivered at our primary comprehensive cancer center. Variables were collected as a part of routine care by the clinical teams caring for the patients and extracted as part of our secondary data analysis.

### 2.4. Statistical Analysis

Descriptive statistics for all patient demographic and clinical information are reported by race and compared using Fisher's exact tests for categorical variables or Wilcoxon rank sum tests for continuous variables. Log-binomial regression modeling estimated differences by race, both unadjusted and adjusting for potential confounders, including age, insurance type, marital status, employment status, median income, educational level, and Charlson comorbidity score. The Kaplan–Meier method was used to estimate time from metastatic diagnosis to medication prescribing, with log-rank tests comparing curves. *p* values of 0.05 or less were deemed to be statistically significant. Analyses were run using SAS statistical software Version 9.4 (Cary, NC).

## 3. Results

### 3.1. Cohort Description

**Figure 1:**
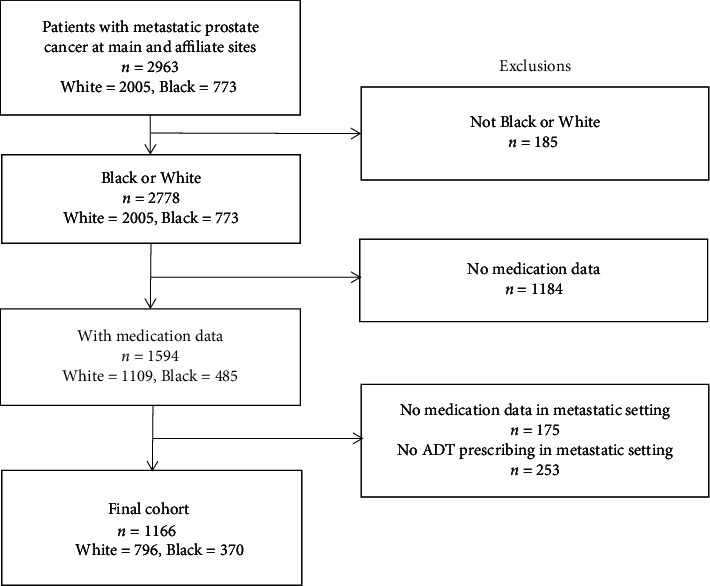
Consort diagram for cohort definition. This consort diagram illustrates how the study cohort was defined, starting with patients with a diagnosis of metastatic prostate cancer at main and affiliate sites (*n* = 2963), then limiting to those who identified as either Black or White (*n* = 2778), as well as those who had medication data (*n* = 1594). This resulted in a final cohort of *n* = 1166.

Over the 5-year study period, 2963 patients were diagnosed with metastatic prostate cancer and seen at UNC main campus or its affiliates. After excluding patients who did not identify as either White or Black, 2778 patients remained, of which 773 (28%) were Black. From here, only patients with medication prescribing data in our EHR were included, leaving 1594 patients (485 Black and 1109 White). To ensure patients were receiving prostate care within our system, we limited our final cohort to those with evidence of ADT medication prescribing after the time of metastatic prostate cancer diagnosis, with our final study population constituting 1166 patients, 370 Black (31.7%) and 796 White (68.3%) (Figure 1).

Differences between Black and White men with prostate cancer in our cohort are noted in Table 1. Black men were younger on average (74.3 years vs. 76.2 years, *p* < 0.01), more likely to be Medicaid insured (7.6% vs. 1.5%, *p* < 0.01) or disabled (13.1% vs. 3.5%, *p* < 0.01) and less likely to have an active MyChart account (electronic portal access) (34.9% vs. 48.5%, *p* < 0.01). Urban/rural residence was similar by racial group, but Black men were more likely to come from geographic areas with low educational achievement (15% vs. 10%, *p* < 0.01) and a lower median income ($55,320 vs. $70,934, *p* < 0.01). Among the total cohort, over 25% were diagnosed with metastatic disease in 2015, with decreasing numbers per year up to 2020 (11%). Most patients were diagnosed with de novo metastatic disease (76.5%) as opposed to progression from documented early-stage disease.

### 3.2. Prostate Cancer Treatment

There were no significant differences in prescribing of any of the systemic agents we evaluated (Table 2). ARPIs (abiraterone, enzalutamide, apalutamide, or darolutamide) were prescribed in 55.8% of White men and 54.9% of Black men (RR: 0.98, 95% CI: -0.13, 0.09). In addition, second-line therapies did not differ by race among patients who received ARPI. Bone protection agents were prescribed in 31.6% of Black men and 36.6% of White men (RR: 0.87, 95% CI: 0.73, 1.03). Chemotherapy was ordered for 16.2% of Black men and 15.6% of White men (RR: 1.04, 95% CI: 0.78, 1.38). PARP inhibitors were prescribed in 1.6% of Black men and 2.4% of White men (RR: 0.68, 95% CI: 0.27, 1.69). First-generation antiandrogens were prescribed in 44.3% of Black men and 41.7% of White men (RR: 1.06, 95% CI: 0.92, 1.22). Other agents, including Radium-223 and sipuleucel-T were prescribed in 11.4% of Black men and 10.4% of White men (RR: 1.09, 95% CI: 0.77, 1.54).

In adjusted, multivariable models, no significant differences were seen in ARPI prescribing. The adjusted relative risk (aRR) for ARPI prescribing between Black and White men was 0.93 (95% CI: 0.81, 1.06). We also did not observe differences between Black and White men on adjusted analysis for first-generation antiandrogens (aRR 1.11; 95% CI 0.94, 1.30). For chemotherapy, PARP inhibitors, and other agents, there were not enough events to run the fully adjusted models. The one difference we observed was in the prescribing of bone protection agents, with an aRR of 0.74 (95% CI: 0.61, 0.90).

### 3.3. Temporal Trends in Treatment

Time from metastatic diagnosis to treatment did not differ within any of the drug types. ARPIs were prescribed after a median of 25.3 months (95% CI: 21.4, 29.3) for Black men and 30.0 months (95% CI: 22.1, 34.3) for White men, with overlapping confidence intervals. Bone protection was prescribed after a median of 70.4 months (95% CI: 57.1, NR) for Black men and 64.5 months (95% CI: 50.8, 75.4) for White men.

**Figure 2:**
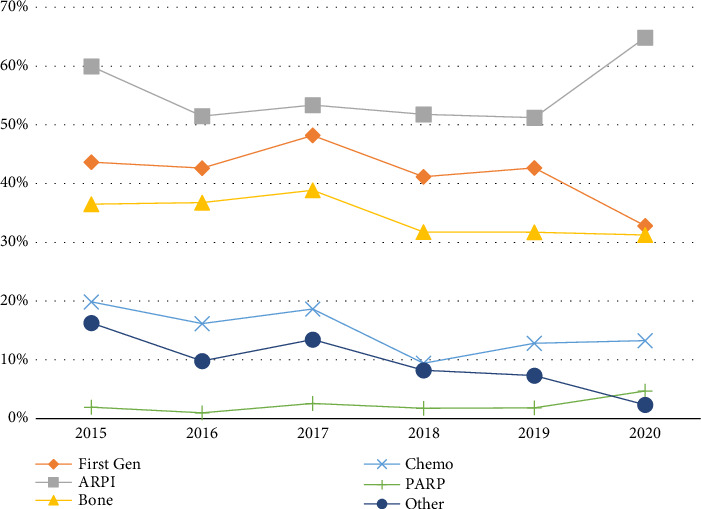
Proportion with receipt of metastatic prostate cancer drug classes over time. First Gen = first-generation antiandrogens, ARPI = androgen receptor pathway inhibitor, Bone = bone protection agents, Chemo = chemotherapy, PARP = poly (ADP-ribose) polymerase, and Other = sipuleucel-T and Radium-223. This line graph shows the proportion of patients who received different metastatic prostate cancer drug classes between 2015 and 2020.

The frequency of ARPI prescribing did not dramatically change in the years prior to FDA approval (55.7%) compared to the year of approval (2018) and beyond (55.2%), without notable differences by race within each time period (Figure 2).

### 3.4. PN

We also evaluated rates of contact with our PN program. We identified chart documentation of contact from PN among 32.6% of the total cohort, 34.3% of Black men, and 31.8% of White men. In the entire cohort, 53.2% of those with contact from PN were prescribed ARPI, compared to 56.6% of those who did not have contact from PN, which was not statistically significant (*p*=0.27). This pattern of ARPI prescribing was held across racial groups. For Black men, 48.8% with PN contact received ARPI versus 58.0% of those without PN contact (*p*=0.09), and for White men, these proportions were 55.3% versus 56% (*p*=0.86).

## 4. Discussion

Here, we present a comprehensive analysis of medication prescribing for advanced prostate cancer within a single health system. In our evaluation of EHR data, we did not observe racial disparities in the prescribing of guideline-recommended therapies for advanced prostate cancer. The only statistically significant difference between Black and White men was in the prescribing of bone protection agents in our fully adjusted model. This may represent a racial disparity in prostate cancer treatment, but it could also represent differences in the treatment of osteoporosis, which is much more common in White compared to Black adults (approximately 12.9% vs. 6.8%) [13]. Without data on hormone sensitivity or diagnoses of impaired bone density, we are unable to determine what is driving this gap within this analysis. We saw a higher prevalence of social risk factors among Black patients, who were living in areas with substantially lower median incomes, with a higher proportion on Medicaid insurance or disabled. We observed lower rates of electronic portal access among Black patients, potentially reducing their ability to communicate with the care team, and higher Charlson scores, indicating more baseline chronic disease. Despite these population differences, medication prescribing by drug class (Table 2) and individual agents did not differ between Black and White patients in our cohort.

We did observe notable findings worth mentioning, with over 40% without documented prescribing of ARPIs, and relatively low utilization of bone protection, chemotherapy, and PARP inhibitors, despite studies showing the benefits of these therapies in appropriately selected patients. These patterns closely mirror those seen in early publications from the IRONMAN registry, which observed 61.7% receiving ARPI, 17.5% receiving chemotherapy, and 1.5% receiving PARP inhibitors [14]. Thus, our findings are quite consistent with prior literature on the delivery of high-quality, evidence-based, systemic prostate cancer therapies. However, in this cohort, we cannot evaluate whether these represent gaps in recommended therapies, given the lack of data on hormone-sensitive versus castrate resistant status within structured EHR variables.

First, it is important to acknowledge that racial disparities are complex and multifactorial, driven by systemic racism but difficult to precisely attribute and mitigate. Insurance coverage is one contributing factor, although numerous studies have shown persistent disparities in treatment delivery and mortality among patients with Medicare, even when stratified for those with additional Part D coverage [8, 15]. Medical mistrust is increased among Black patients, in large part due to historical mistreatment of Black patients by medical institutions [16]. Perceptions of risk, cost, and burden of treatment differ by race [17, 18]. Higher levels of mistrust and lower quality communication have independently been associated with reduced uptake of guideline-recommended care in several cancer types [19, 20]. As noted above, a recent SEER-Medicare analysis observed disparities in treatment for advanced prostate cancer for Black men compared to their White counterparts, findings consistent with prior studies of surgical treatment for early-stage disease.

How then were these racial disparities in care delivery not observed within our cohort? Our health system has developed several initiatives to emphasize population health and specifically racial equity in care delivery. We have a robust cancer navigation program, which performs regular outreach to new and existing patients receiving cancer treatment at our clinics [21]. This involves proactive identification of barriers to care, whether transportation, financial, beliefs, or otherwise. Several studies have shown the benefits of navigation on receipt of recommended cancer care delivery [22–24]. Given this, we performed analyses to evaluate contact with our navigation program and any effect on medication prescribing. We observed high rates of contact with this program, approximately a third of our entire cohort. However, the rates of contact did not differ significantly between Black and White patients, nor did medication prescribing differ for patients who did or did not have contact with this program. There may be unquantifiable benefits to underserved populations by instituting a program focused on identifying and resolving barriers to cancer care, and from our view, this is the right thing to do for both individual and population health.

Another potential explanation for the equitable care delivery we observed is the health system's investment in our institutional specialty pharmacy. There are several facets to the cancer pharmacy team, including specialty pharmacists who are familiar with the clinical care teams, the Medication Assistance Program (MAP), and the clinic-based clinical pharmacist practitioners (CPPs). Our MAP team operates across the main campus and affiliate sites, performing proactive benefits investigations, completing prior authorizations, and securing funding through grants or manufacturer assistance programs whenever appropriate. The MAP team assists with obtaining financial information and completing forms to secure funding for patients to the extent that they are able to. They additionally perform price comparisons via direct-to-consumer specialty pharmacies, such as CostPlus drugs, to give patients access to needed cancer drugs at the lowest cost. Our healthcare system also has specialty pharmacists and CPPs for tailored patient education and side effect management, which may improve adherence, tolerance, and patient understanding of treatment regimens [25]. It is difficult to quantify contact with pharmacy services including institutional specialty pharmacy, MAP program, and CPP through the EHR; thus, it is difficult to say whether this is the driver of the equitable care we observed. However, the idea that providers have confidence that patients will receive ample support to get affordable access to drugs, along with regular support and education along the way, is certainly a possible contributor to equitable care delivery. Prior research has suggested that insurance limitations and financial hardship both weigh heavier on Black patients compared to White patients [26]. A comprehensive pharmacy support program may help mitigate those concerns and expand access. This was not adequately evaluated in the current research effort but merits further study.

Lastly, while our analysis focused on racial disparities, there may be other disparities present in cancer care delivery in our health system that merit further study. Urban compared to rural individuals are known to face different access to care, leading to gaps in early-stage prostate cancer care [27]. In addition, insurance is generally prevalent in prostate cancer studies, given the predominance of Medicare coverage in a disease that largely affects older adults. However, differences in Part D coverage and/or low-income subsidies may have an outsized effect on prescribing and access to these evidence-based therapies. These patterns are more nuanced but have been seen across several other cancer types, while Part D costs remain quite high for several antiandrogens [28–30]. Thus, while we are reassured by the lack of racial disparities, there are potentially other important sociodemographic gaps that need to be explored.

We should note several limitations of this study. It was conducted within a single health system and limited to retrospective EHR data only, lacking claims or cancer registry inputs to externally validate cancer treatments, diagnosis, and stage, thus limiting generalizability to other health systems. Our decision to exclude patients who did not have evidence of medication prescribing or ADT within our system limits our cohort to a dataset with complete documentation of key outcomes within our records but may miss out on individuals receiving care as a second opinion, coming from a distance, or navigating care across health systems. These are important groups to consider in future analyses. Notably, we would have also missed out on those who were not prescribed ADT by clinicians in our network, despite clinical guidelines to do so. However, as a sensitivity analysis, we evaluated for differences in ADT prescribing by race among these excluded patients, and we did not see any racial disparities. Thus, this added inclusion criterion likely did not alter our primary finding.

## 5. Conclusions

In a comprehensive analysis of care delivery for advanced prostate cancer within a single health system, we did not observe racial disparities in prescribing. Our institution offers several population health interventions that may have mitigated potential disparities, including PN and specialty pharmacy services aimed at ensuring access and minimizing patient financial burdens. Further work should explore disparities in urban–rural and Part D insurance coverage and better characterize the roles that PN and pharmacy assistance programs play in prostate cancer medication access.

## Figures and Tables

**Table 1:** Patient demographics and clinical characteristics.

Characteristic	Total *n* = 1166		*p* value
	Black (*n* = 370, 31.7%)	White (*n* = 796, 68.3%)	
Patient level demographics			
Mean age (SD)	74.28 (10.19)	76.20 (9.34)	< 0.01
Insurance			< 0.01
Private	75 (20.3%)	147 (18.5%)	
Medicare	247 (66.8%)	609 (76.5%)	
Medicaid	28 (7.6%)	12 (1.5%)	
Self-pay	20 (5.4%)	28 (3.5%)	
Married (%)	205 (56.0%)	581 (73.7%)	< 0.01
Employment status			< 0.01
Employed (%)	43 (11.6%)	119 (14.9%)	
Retired (%)	239 (64.6%)	599 (75.3%)	
Disabled (%)	47 (12.7%)	27 (3.4%)	
Other (%)	41 (11.1%)	51 (6.4%)	
Median Charlson Index (IQR)	4 (3–6)	4 (2–5)	< 0.01
Active MyChart account (%)	129 (34.9%)	386 (48.5%)	< 0.01
Zip code level characteristics			
RUCA category			
Metropolitan (%)	280 (75.7%)	617 (77.6%)	0.40
Micropolitan (%)	47 (12.7%)	80 (10.1%)	
Small town/rural (%)	43 (11.6%)	98 (12.3%)	
Regional educational achievement^∗^, average (SD)	15% (0.09%)	10% (0.07%)	< 0.01
Median income, average (SD)	$55,320.19 ($23,665.36)	$70,934.92 ($28,817.51)	< 0.01
Treatment characteristics			
Surgery (%)	20 (5.4%)	59 (7.4%)	0.26
Radiation (%)	103 (27.8%)	212 (26.6%)	0.67
Metastatic year of diagnosis			
2015 (%)	109 (29.5%)	198 (24.9%)	0.02
2016 (%)	54 (14.6%)	150 (18.8%)	
2017 (%)	48 (13.0%)	145 (18.2%)	
2018 (%)	52 (14.1%)	118 (14.8%)	
2019 (%)	64 (17.3%)	100 (12.6%)	
2020 (%)	43 (11.6%)	85 (10.7%)	
De novo metastatic (%)	284 (76.8%)	608 (76.4%)	0.94
Metastatic within 1 year of early-stage diagnosis^+^	71 (82.6%)	144 (76.6%)	0.34
Utilizing patient navigation within 6 months of diagnosis (%)	127 (34.3%)	253 (31.8%)	0.42
Mean number of navigation contacts within 6 months of diagnosis (SD)	4.55 (4.47)	4.04 (3.42)	0.36

*Note:* This table shows patient demographics and clinical characteristics across Black versus White patients. The table presents characteristics at the patient level, zip code level, and treatment level.

^∗^Educational achievement is defined as the proportion of males under 25 years old who did not complete high school; thus, a lower value indicates lower education in that zip code.

^+^Denominator is patients who did not have de novo metastatic disease.

**Table 2:** Proportion of Black and White patients receiving guideline-recommended therapies for metastatic prostate cancer.

Medication	Black (*n* = 485)	White (*n* = 1109)	*p* value^∗^	RR^+^ (95% CI)
Androgen receptor pathway inhibitor	203 (54.9%)	444 (55.8%)	0.77	0.98 (0.13, 0.09)
Bone protection	117 (31.6%)	291 (36.6%)	0.10	0.87 (0.73, 1.03)
Chemotherapy	60 (16.2%)	124 (15.6%)	0.78	1.04 (0.78, 1.38)
PARP inhibitors	6 (1.6%)	19 (2.4%)	0.40	0.68 (0.27, 1.69)
First-generation antiandrogen	164 (44.3%)	332 (41.7%)	0.40	1.06 (0.92, 1.22)
Other (Radium-223 and sipuleucel-T)	42 (11.4%)	83 (10.4%)	0.63	1.09 (0.77, 1.54)

*Note:* This table outlines the proportion of Black and White patients receiving guideline-recommended therapies, including androgen receptor pathway inhibitors, bone protection, chemotherapy, PARP inhibitors, first-generation antiandrogens, and others.

^∗^ *p* values were calculated using Fisher's exact test for any prescribing of the medication category since the time of metastatic diagnosis versus no evidence of prescribing.

^+^Relative risk, unadjusted, as adjusted results were not available for all drug categories.

## Data Availability

The data that support the findings of this study are available on request from the corresponding author. The data are not publicly available due to privacy or ethical restrictions.

## Supporting Information

The supporting table includes an overview of specific medications within each of the categories of medications of interest.
