# Supplementary material for: Racial Disparities in the Prescribing of Guideline-Recommended Medications for Metastatic Prostate Cancer: A Retrospective Cohort Study
Source: Prostate Cancer. 2025 Nov 26;2025:6500084. doi: 10.1155/proc/6500084 (PMC12674856; doi:10.1155/proc/6500084)
Supplement: Supporting Information — Additional supporting information can be found online in the Supporting Information section. [file 6500084.f1.docx]

**Supp Table – Medications:**

/* Anti-androgen drugs*/

ABIRATERONE

BICALUTAMIDE

CASODEX

FLUTAMIDE

APALUTAMIDE

ERLEADA

ENZALUTAMIDE

DAROLUTAMIDE

NUBEQA

XTANDI

ZYTIGA

YONSA

/* ADT*/

LEUPROLIDE

LUPRON

DEGARELIX

FIRMAGON

ELIGARD

GOSERELIN

RELUGOLIX

/* PARP*/

LYNPARZA

OLAPARIB

RUCAPARIB

RUBRACA

/* bone protection*/

DENOSUMAB

XGEVA

ZOLEDRONIC ACID

PROLIA

/* chemo*/

CABAZITAXEL

DOCETAXEL (TAXOTERE) IVPB (OVF)

/* other*/

SIPULEUCEL

RA-**223**

RADIUM RA **223**

Xofigo
